# Empowerment of nurses for integrating clients’ religion/spirituality into clinical practice: outcomes of an online training program

**DOI:** 10.1186/s12912-021-00723-y

**Published:** 2021-10-25

**Authors:** Hasan Amiri, Jamileh Farokhzadian, Batool Tirgari

**Affiliations:** 1 Department of Community Health Nursing, Razi Faculty of Nursing and Midwifery, Kerman University of Medical Sciences, Kerman, Iran; 2 Nursing Research Center, Kerman University of Medical Sciences, Kerman, Iran

**Keywords:** Education, Practice, Religion, Nurses, Spirituality, Spiritual care

## Abstract

**Background:**

Integration of clients’ religion/spirituality (R/S) into nursing practice can have effective outcomes in clients’ health. In this regard, nurses’ lack of competency can disrupt this process and interfere with the treatment process. Limited studies examined the impact of training programs on nurses’ competency in spiritual care and integration of clients’ R/S into clinical practice. This study aimed to investigate the impact of an online training program on nurses’ empowerment for integrating clients’ R/S into clinical practice.

**Methods:**

In the present interventional study, 80 nurses were selected by stratified sampling from two hospitals in the southeastern Iran. Nurses were randomly divided into the intervention (*n* = 40) and control (*n* = 40) groups. An online training program was performed for the intervention group in four 2-hour sessions during three weeks. Data were collected from all participants using the R/S Integrated Practice Assessment Scale (RSIPAS) before and one month after the intervention.

**Results:**

Prior to the intervention, scores of integrating clients’ R/S into clinical practice were not significantly different between the intervention and control groups (t = 0.23, *p* = 0.81). However, after the training program, these scores increased significantly with a very large effect size compared to the control group (t = 4.31, *p* = 0.001). Although the control group scores improved significantly after the intervention compared to the pre-intervention stage, the effect size was very small (t = -2.55, *p* = 0.01).

**Conclusions:**

The online training program had a positive effect on nurses’ competency for integrating clients’ R/S into clinical practice in the intervention group. Due to the importance of integrating clients’ R/S into clinical practice, nurses’ competency should be strengthened in this area. Managers are suggested to consider appropriate strategies in order to empower nurses in integrating clients’ R/S into clinical practice. Nurse educators can benefit from our experiences in application of online training programs in nursing schools.

## Background

Religion/ spirituality (R/S), as a key component for understanding human development and behavior, has long been an integral part of human existence [1, 2]. Religion is considered as a set of shared beliefs and practices among members of a community to worship God. Spirituality is determined as an individual’s search to reach answers about ultimate goal in life, its meaning, and relationship to the sacred or transcendent. This process can be considered as the causative factor of religious rituals in forming a community [3, 4]. Religion and spirituality share concepts such as feelings, thoughts, experiences, and behaviors in searching for the sacred. Although religion and spirituality differ conceptually, they are applied interchangeably since they are often interconnected in practice [5].

Since holistic nursing targets the physical, mental, social, and spiritual needs of the people’s lives, religion and spirituality should be highly considered by nurses to provide holistic care [5, 6]. Clients prefer that healthcare providers be aware of their R/S beliefs, involve these beliefs in their course of treatment, and talk about the impact of R/S beliefs on the healing process [7]. Evidence-based practice (EBP), as one of the most widespread approaches to the decision-making process in nursing, integrates the best clinical research evidences and nurses’ expertise into patients’ preferences and values (such as R/S). Therefore, integration of clients’ R/S into the clinical practice by nurses shows their efforts to implement EBP [7, 8].

Studies show that integration of clients’ R/S into clinical practice and provision of spiritual care by healthcare providers can improve physical and mental health outcomes for clients. For example, such integrations can increase patients’ spiritual well-being, quality of life [9], hope [10], but reduce stress, anxiety, depression [11–13], and sense of loneliness [14]. Although nurses are aware of the importance of patients’ R/S as an important part of holistic and patient-centered care, they frequently ignore R/S needs and mostly focus on patients’ physical care [15, 16]. Studies in different countries [7, 8, 17, 18] and Iran [15, 16, 19, 20] show that nurses ignore integration of the clients’ R/S into clinical practice for various reasons, including non-competence, lack of time, underestimation of R/S, and inadequate training in this regard. These studies also indicate the need for further studies on the empowerment of nurses to provide R/S care. Despite the emphasis of studies, researchers believe that limited efforts have been made to design continuing education programs to empower nurses in integrating clients’ R/S into clinical practice. However, more evidence-based interventions are needed in different cultures and settings. Ethical codes and accreditation standards have also addressed training nurses to integrate clients’ R/S into their practices ethically and effectively [7, 8, 21].

Several studies implemented training programs to improve competency in the provision of spiritual care among nurses and nursing students, reporting that the participants’ spiritual care competency increased after the educational programs. As they concluded, further educational programs are required for healthcare providers in order to provide R/S services to clients [15, 22–25]. Another study demonstrated that curricular developments should focus on R/S diversity in various countries. Nursing education should prepare nurses to address the positive role of R/S effectively in the well-being of indigenous people throughout the world. Since many countries are affected by the unprecedented rise in international migration, cultural competencies are required to effectively address R/S needs of diverse newcomers [2].

Given that in a multicultural context, such as Iran, R/S is an integral part of the cultural identity, nurses should benefit from paying attention to R/S to develop their competency for integrating clients’ R/S into clinical practices. Furthermore, studies are limited over the effectiveness of continuing education and training programs in increasing competency and empowering nurses for integration of clients’ R/S into clinical practice. As a result, further rigorous interventional studies are needed to develop continuing training programs for integrating clients’ R/S into clinical practice and evaluating outcomes of these programs in different contexts and cultures. Therefore, this study aimed to evaluate the effectiveness of an online training program in empowering nurses for integrating clients’ R/S into clinical practice.

## Methods

### Study design and settings

The present educational intervention was conducted to empower nurses in order to integrate clients’ R/S into clinical practice. The study was conducted using a pretest-posttest design using the intervention and control groups. The study setting included Sina and Imam Ali hospitals, as two state-funded hospitals in Zarand City, Kerman Province, in the southeast of Iran.

### Sampling procedure

The study population included all nurses (N = 300) working in the two above-mentioned hospitals. The sample size was calculated according to a pervious study and the sample size formula. In this regard, the required sample size was calculated as 40 participants in each study group (a total of 80 participants) by taking into account α = 0.05, test power of 80 %, and a moderate effect size (Cohen d = 0.5). The study participants were selected using stratified random sampling method, so that equal numbers of nurses were employed from each hospital based on the lists of nurses working in the studied hospitals. Given the almost equal number of nurses working in the two hospitals, 40 nurses were selected from each hospital via the random number Table (20 nurses for the intervention and 20 nurses for the control groups). As a result, 80 nurses were assigned into the intervention (n = 40) and control (n = 40) groups. Based on the inclusion criteria, nurses with bachelor or master’s degree, and with at least six months of work experience were included in the study. The exclusion criteria were lack of attendance in the training program for more than one session and exposure to major stressors such as death of a family member or friend, divorce, etc. In the present study, none of the nurses was excluded based on the inclusion criteria.

### Measurement

To collect the study data, two instruments were applied. The participants’ demographic information was gathered through a questionnaire consisting of 11 items (Table 2).

The Religious/Spiritually Integrated Practice Assessment Scale (RSIPAS) was administered to measure the practitioners’ competence and overall orientation in integrating clients’ R/S into practice [8, 26]. The RSIPAS includes four subscales and 40 items: (a) self-efficacy in integrating clients’ R/S into practice (13 items), (b) attitudes towards integrating clients’ R/S into practice (12 items), (c) perceived feasibility to engage in R/S integrated practice (6 items), and (d) behaviors associated with integrating clients’ R/S into practice (9 items). The participants were required to answer the RSIPAS items on a 5point Likert scale using the “strongly disagree, disagree, neutral, agree, and strongly agree” options (in the first three subscales) and “never, rarely, sometimes, often, and very often” options for the behaviors subscale. Three items included negatively worded stems (item 12 in attitude subscale as well as items 3 and 4 in feasibility subscale). These items were reversely scored in inferential analyses. Possible attainable scores can range from 40 to 200, so that higher scores indicate higher levels of competence in integrating clients’ R/S into practice. The RSIPAS content validity was confirmed according to the opinions of experts. Its criterion, construct, discriminant, and factor analysis validity were also investigated and determined. Moreover, reliability of RSIPAS was confirmed with an overall Cronbach’s alpha of 0.95. Internal consistency of self-efficacy (α = 0.91), attitudes (α = 0.88), behaviors (α = 0.87) perceived feasibility (α = 0.88) subscales were corroborated [8].

In order to use RSIPAS, official permissions were received from the original designers through Email. To customize this scale for Iranian culture, the original RSIPAS was translated precisely into Persian (forward translation). The backward translation of the Persian version was performed by a proficient English translator. Later, the translated version was compared with the original version and its content validity was corroborated by 10 nursing faculty members. The overall reliability of the Persian version of RSIPAS was confirmed by Cronbach’s alpha of 0.91. The Cronbach’s alpha values calculated for self-efficacy, attitudes, and behaviors subscales related to integrating clients’ R/S into practice as well as perceived feasibility to engage in R/S integrated practice were 0.89, 0.81, 0.76, and 0.91, respectively.

### Data collection

Followed by receiving the Code of Ethics and the necessary permissions from the hospital authorities, data were collected using an anonymous, self-reported, and electronic questionnaire from November to February 2021. To this end, WhatsApp groups were created for the intervention and control groups by the first researcher. Nurses who enrolled in the study were asked to sign the informed consent forms containing the study purpose and process two weeks prior to the training program. The participants were also ensured that their participation in study was completely voluntary and their decision to continue or decline the study process would not cause negative impacts on their occupational status. Furthermore, confidentiality of their information was warranted. At the pre-test and post-test (one month after the intervention), the questionnaire’s link was sent to WhatsApp groups and the participants were instructed on completing the questionnaires. Initially, the online survey provided the respondents with the study purpose and definitions of religion and spirituality. To obtain the highest response rate, appropriate time was allocated on data collection and a deadline was set to submit the completed questionnaires for the participants. Moreover, participants were provided with detailed information about the training program and ensured to receive a valid education certificate upon completing the educational course. The first researcher set the time of educational sessions with the participants in the intervention group and sent them reminders (in WhatsApp and SMS) to participate in the sessions at the scheduled times.

### Description of the training program

The curriculum content was designed and developed based on the related literature over competencies to effectively integrate clients’ R/S into nursing practice [3, 7, 8, 15, 25, 27–30]. The intervention aimed to improve burses’ confidence and competence in the face of R/S issues in their clinical practices. The training program was presented online to increase the nurses’ participation, accessibility, convenience, and health safety during the COVID-19 outbreak. The program was free of charge and eligible nurses received an education certificate upon completion of the course. The online training program was delivered through Sky Room platform in eight hours (4 sessions of 2 h) within three weeks, through the basic competency contents were covered comprehensively. The program directors (JF and BT) presented the contents via lecture, demonstration, question and answer, PowerPoint presentations, hands-on exercise, group discussion, video film, case report, and scenario. Moreover, members of the intervention group had offline access to the program materials and were provided with the recorded videos and audio files of the course through the cyberspace. The topics presented in the program are shown in Table 1. While intervention group was provided with educational materials, the control group did not receive such educational contents.

**Table 1:** Educational contents related to integrating clients’ R/S into clinical practice (intervention group)

Session 1	- Introduction and explanation of the goals and expectations related to the program - The concept of holism, holistic care, and its importance in nursing - R/S concepts; differences and similarities - The importance of integrating client’s R/S in to clinical nursing practice; its outcomes for clients and healthcare providers - How do nurses’ spiritual beliefs affect their life style, response to disease, and healthcare & treatment choices - Some discussions about finding life meaning and goal in the face of suffering, disease, and death in addition to concepts such as trust and honesty - Definition of spiritual care, history and condition of spiritual care in Iran and the world - Exercise: Nurses were asked to provide scenarios regarding their clinical experiences for the next session.
Session 2	- Self-consciousness and self-evaluation, identification of limitations and evaluation of the nurses’ skills with regard to integrating client’s R/S into nursing practice - Evaluation and integration of the client’s R/S into the nursing practices - Identification of the current empirically supported methods for integrating clients’ RS in practice. Such methods include standardized assessment tools, manualized interventions, empirically supported practical behaviors related to client’s characteristics and preferences -Evaluation of the client’s R/S needs and implementation of strategies for receiving a spiritual history from the clients - Considering the patient’s spiritual needs and respect for the client’s R/S beliefs - Evaluation of the client’s reactions to feelings of loneliness, weakness, disease, and death - Collection of information about the clients’ spiritual and religious beliefs -Introduction of some appropriate questions to ask if you wanted to learn more about a client’s spiritual and religious beliefs/values - Distinction between helpful and harmful types of spirituality - Provision of a model to evaluate the patients’ R/S and provision of some strategies to collect information about the clients’ R/S history - Recognition of positive and negative mechanisms and strategies of the clients regarding R/S - Recognition of the spiritual sources of clients and hospitals - Examples of questions to evaluate clients’ R/S - Exercise: discussion about the scenarios, R/S evaluation in the scenarios suggested by nurses, application of the questionnaires and the model suggested by instructors
Session 3	- Recognition of R/S problems of the clients (nursing diagnosis) such as spiritual distress - Spiritual distress indicators and verbal statements of the clients with regard to spiritual distress - Use of verbal/non-verbal skills related to the clients’ culture - Communication skills with respect to spiritual care within the context of patients’ relationship with nurses and the multidisciplinary team (spiritual assessment and spiritual support) -Transfer of bad news and terminal patients and their spirituality: related points to be considered” - Recognition of R/S conflicts, personal needs of patients in relation to R/S - Planning for implementation of R/S care - Recognition of positive and negative R/S coping strategies - Interventions to reduce spiritual distress, decrease R/S problems, and promote spiritual growth of the client - Provision of R/S sources and advisors for the client and support of his/her R/S rituals - Introduction of concepts such as forgiveness, thankfulness, mindfulness, presence, hope, meaning, connection to transcendent power, spiritual change, and ultimate reality - Skills necessary for the nurse to provide R/S interventions - Introduction and review of two clinical guides localized for spiritual care in Iran - Exercise: determining problems in spiritual diagnoses and scenarios as well as designing interventions for such problems
Session 4	- Evaluation of the outcomes of R/S care for the clients - Application of the evaluation methods for the R/S interventions - Documentation of the nursing process in spiritual care and integration of the client’s R/S into nursing practice - Limitations and barriers of the R/S care - Reflection on the personal experiences related to aspects of spiritual care in nursing practice - Planning for client’s discharge - Summary and conclusion of the program - Exercise: review of the scenarios designed in previous sessions, nursing process, and evaluation of the R/S care.

### Statistical analysis

To analyze the data, SPSS 21 (SPSS Inc., Chicago, Ill., USA) was used. Descriptive statistics (frequency, percentage, mean, and standard deviation) were applied to describe the background characteristics of nurses. Chi-square and Fisher’s exact tests were also run to compare similarity of the intervention and control groups in terms of background variables. According to the normality test (the Kolmogorov-Smirnov), independent samples t-test was used to compare the scores of RSIPAS between the two groups at the pre- and post-intervention stages. Paired t-test was also administered to compare the RSIPAS scores in each group at pre- and post-intervention stages. Analysis of covariance was applied to control the impact of pretest on the scores of RSIPAS. The significance level was set at P < 0.05.

## Results

### Baseline background information of the participants

All participants completed the training program and questionnaire (response rate = 100 %). The results showed that most of the participants in the intervention and control groups were married (100 %), female (87.5, 97.5 %), 30–40 years (55.0, 72.5 %), and had bachelor’s degree (100, 92.5 %) with a work experience of 10–20 years (45.0, 52.5 %). In addition, they mostly were nurses (97.50, 92.50 %) working in the general wards (65.0, 55.0 %). Most of the participants were moderately religious (82.5, 70.0 %), were moderately spiritual (67.5, 65.0 %), did not receive prior training in R/S (85.0, 95.0 %), and did not use a specialist in providing R/S care (97.5, 95.5 %). The two study groups were homogenous in terms of their background information (Table 2). At the baseline, the intervention and control groups indicated no statistically significant differences in their total scores of integrating clients’ R/S into clinical practice and across the subscales of the RSIPAS. Independent samples t-test indicated homogeneity of the participants in the two study groups at the baseline (Table 3).

**Table 2:** Comparison of the background information between the two groups

Groups		Intervention		Control		Statistic test	*P*-value
Variables		n	%	n	%		
Age groups	< 30	3	7.5	2	5.0	2.66^a^	0.26
	30–40	22	55.0	29	72.5		
	> 40	15	37.5	9	22.5		
Gender	Male	5	12.5	1	2.5	2.88^b^	0.09
	Female	35	87.5	39	97.5		
Level of education	Bachelor	40	100.0	37	92.5	3.11^b^	0.08
	Master	0	0	3	7.5		
Work position	Nurse	39	97.5	37	92.5	2.05^b^	0.36
	Head nurse and supervisors	1	2.5	3	7.5		
Work experience (years)	˂10	18	45.0	13	32.5	1.43^a^	0. 49
	10–20	18	45.0	21	52.5		
	> 20	4	10.0	6	15.0		
Type of ward	Emergency	10	25.0	5	12.5	13. 63^a^	1.14
	Critical (CCU, ICU, NICU, Dialysis)	4	10.0	11	27.5		
	Internal medicine, Palliative care, Nerves, Obstetrics and Gynecology, pediatric, Surgery	26	65.0	22	55.0		
	Supervisory	0	0	2	5		
Degree of religiosity	Very Religious	3	7.5	5	12.5	2.05^b^	0.56
	Moderately religious	33	82.5	28	70.0		
	Slightly religious	3	7.5	4	10.0		
	Not religious	1	2.5	3	7.5		
Degree of spirituality	Very spiritual	6	15.0	6	15.0	0.09^a^	0.95
	Moderately spiritual	27	67.5	26	65.0		
	Slightly spiritual	7	17.5	8	20		
	Not spiritual	0	0	0	0		
Attendance at prior training in R/S	Yes	6	15.0	2	5.0	2.22^b^	0.14
	No	34	85.0	38	95.0		
Use of a specialist in providing R/S care	Yes	1	2.50	1	2.5	0.0001^b^	1
	No	39	97.5	39	95.5		

^a^ Chi-square test

^b^ Fisher’s exact test

**Table 3:** Comparison of the scores of integrating clients’ R/S into clinical practice (RSIPAS) between the control and intervention groups before and after the training program

Variables	Time	Before the Intervention	After the Intervention	Within group differences	ES^*^ (Cohen d)	Paired t-test	*P*- value
	Groups	M ± SD	M ± SD				
Self-efficacy	Intervention	39.46 **±** 6.27	45.23 **±** 4.71	5.76	1.04	-4.60	**0.001**
	Control	40.82 **±** 8.74	41.97 **±** 6.08	1.15	0.15	-1.61	0.11
	Independent t-test	-0. 54	2.64				
	*P*-value	0.59	**0.01**				
	ES^*^ (Cohen d)	0.17	0.59				
Attitudes	Intervention	43 **±** 6.46	44.15 **±** 3.97	1.15	0.21	-1.23	0.22
	Control	41.02 **±** 7.76	41.65 **±** 4.98	0.62	0.09	0.74	0.46
	Independent t- test	1.23	2.47				
	*P*-value	0.22	0.01				
	ES^*^ (Cohen d)	0.27	0.55				
Perceived feasibility	Intervention	16.30 **±** 2.68	18.4 **±** 1.66	2.1	0.94	-5.05	**0.001**
	Control	15.82 **±** 2.91	15.67 **±** 2.92	-0.15	0.05	0.37	0.71
	Independent t- test	0.75	5.12				
	*P*-value	0.45	0.001				
	ES^*^ (Cohen d)	0.17	1.21				
Behaviors	Intervention	22.67 **±** 6.26	30.7 **±** 4.98	8.02	1.41	-9.29	**0.001**
	Control	23.22 **±** 7.77	25.92 **±** 6.71	2.70	0.37	-3.73	**0.001**
	Independent t- test	0.34	3.61				
	*P*-value	0.72	0.001				
	ES^*^ (Cohen d)	0.07	0.80				
Total of RSIPAS	Intervention	121.53 **±** 15.11	138.74 **±** 10.88	17.20	1.30	-7.58	**0.001**
	Control	120.79 **±** 21.82	125.12 **±** 16.42	4.33	0.22	-2.55	**0.01**
	Independent t- test	0.23	4.31				
	*P*-value	0.81	**0.001**				
	ES^*^ (Cohen d)	0.03	0.97				

Bold p-values are significant at level of ≤ 0.05

^*^Effect size (ES): 0-0.2 = small effect, 0.2–0.5 = moderate effect, > 0.5–0.7 = large effect, and > 0.7 = very large effect

### Outcomes

Table 3 shows the level of integrating clients’ R/S into clinical practice and its subscales between the two study groups before and after the intervention. At the beginning of the study, the mean scores of integrating clients’ R/S into clinical practice in the intervention and control groups were 121.65 ± 15.11 and 120.79 ± 21.82, respectively, showing no significant difference between the two groups (t = 0.23, *p* = 0.81). The mean scores of integrating clients’ R/S into clinical practice in the intervention and control groups were 138.74 ± 10.88 and 125.12 ± 16.42, respectively after the intervention, showing a significant difference between the two groups (t = 4.31, *p* = 0.001).

Based on the results of paired- *t* test, the scores of integrating clients’ R/S into clinical practice increased 4.33 and 17.20 points in the control and intervention groups one month after the intervention, respectively. However, magnitude of the difference had a small effect size in the control group (Cohen d = 0.22), but a statistically significant difference was observed in scores of the intervention group with a very large effect size (Cohen d = 1.30).

The covariance analysis test was run to control and investigate the effects of pretest on nurses’ scores of integrating clients’ R/S into clinical practice. The results showed a statistically significant difference between the control and intervention groups in the total post-test scores of integrating clients’ R/S into clinical practice (Table 4). These results are also consistent with the results of Table 3

**Table 4:** Results of covariance analysis for the control and intervention groups

		df	Mean square	F	*P*-value
Self-efficacy	Intercept	2	1971.37	96.90	**0.001**
	Pre-test	1	724.11	35.59	**0.001**
	Group	1	280.39	13.78	**0.001**
	Error	77	20.34		
Attitudes	Intercept	2	1541.27	121.08	**0.001**
	Pre-test	1	606.10	47.61	**0.001**
	Group	1	58.66	4.60	**0.035**
	Error	77	2.72		
Perceived feasibility	Intercept	2	235.65	55.56	**0.001**
	Pre-test	1	113.78	26.82	**0.001**
	Group	1	126.100	29.73	**0.001**
	Error	77	4.24		
Behaviors	Intercept	2	1452.96	82.92	**0.001**
	Pre-test	1	1377.99	78.64	**0.001**
	Group	1	519.85	29.66	**0.001**
	Error	77	17.52		
Total of RSIPAS	Intercept	2	7490.22	85.86	**0.001**
	Pre-test	1	8212.98	94.14	**0.001**
	Group	1	3398.14	38.95	**0.001**
	Error	77	87.23		

Bold p-values are significant at level of ≤ 0.05

## Discussion

The present study evaluated the effectiveness of an online training program in empowering nurses for integrating clients’ R/S in clinical practice. The findings indicated that the training program increased self-perceived competency of the intervention group in integrating clients’ R/S in clinical practice significantly and with a very large effect size. Similar to the present study, several studies reported positive effects of the training programs on spiritual care competencies and integration of clients’ R/S into clinical practice in healthcare providers, including nurses and nursing students [22, 23, 31–33]. Pearce et al. (2019) conducted an interventional online study that increased spiritual competencies and integration of clients’ R/S into practice among mental healthcare providers. In addition, the participants believed that their perceived barriers to integrating client’s R/S into practice reduced after the intervention. The study also collected information about the participants’ satisfaction with the content and format of the program via qualitative methods. According to the findings, participants were highly satisfied with the content and format of the online training program. These results suggest that a new and concisely organized online training program can meet the clinical needs and professional requirements for spiritual competencies considering the lack of continuing education in this cross-cultural area. This study suggests future researchers to examine how to integrate this program and similar programs into complementary studies to empower healthcare providers in terms of religious/spiritual competence and its impact on the clinical care [34].

Yilmaz and Gurler (2014) investigated the impact of spiritual integration into undergraduate nursing programs in Turkey. As they concluded, little attention has been paid to spirituality before the intervention, but R/S integration into the undergraduate nursing curriculum increased their spiritual competencies of the nursing students after the intervention. Therefore, they recommended inclusion of the spirituality courses into the nursing education [24]. Paal et al. (2015) conducted a systematic review over 46 articles to examine the outcomes of spiritual care training courses among healthcare providers. They categorized the study outcomes into three categories, including (1) recognition of spirituality at the individual level; (2) successful integration of spirituality into clinical practice; (3) positive changes in communication with patient and spirituality integration into communicating with patients. This study emphasized that spiritual care training was one of the ways to integrate R/S into holistic patient care among the healthcare professionals. Spiritual care education can also challenge the spirituality gap in the healthcare system. Personal values, emotions, as well as physical and spiritual distress are among the most common barriers, perceived by healthcare professionals to integrate spirituality into practice. In addition to individual stressors, organizational factors such as lack of time and high workload, intra-team problems such as mistrust, and different value systems were reported as barriers to integrating R/S into clinical practice. Our findings also emphasized that application of skills such as increasing the sensitivity of healthcare providers to their spirituality, clarifying the role of spirituality in healthcare, and training students can be effective in integrating clients’ R/S into clinical care. Participants in training programs should evaluate their R/S beliefs by devoting sufficient time before integrating R/S into their professional practices. However, addressing patients’ R/S is not possible without considering the individual beliefs and needs of healthcare providers [35].

At the pre-intervention stage, the scores of integrating clients’ R/S into clinical practice increased significantly in the control group compared with the pre-test, but the effect size was small and insignificant. This change may be due to the effect of confounding variables such as individual beliefs, cultural factors, and unique spiritual perspectives of the participants, which were not addressed in this study. Moreover, this study was conducted among Iranian and Muslim nurses. Given the emphasis of Islam on observance of R/S principles in the healthcare process, providing the necessary conditions for R/S practices and fulfillment of the patients’ religious needs is of great importance. Therefore, the nurses’ responses to RSIPAS in the pre-intervention phase might have affected their answers to the questionnaire after the intervention. A similar study reported that the mean score of spiritual care in the control group increased after integration of spirituality into nursing education programs [24]. However, further studies should be conducted on different groups of healthcare providers by controlling the confounding variables.

## Limitations and strengths

This study had some limitations that need to be observed. A self-report questionnaire was administered to measure effectiveness of an educational program in improving the nurses’ competency in integrating clients’ R/S into clinical practice. Assessment of competency might have been affected by the participants’ bias inherent in the self-report questionnaire. In other words, since nurses may have tended to overrate their levels of competency, the data might not reflect the actual level of nurses’ competency. Future studies can use objective rather than self-reported means of evaluating competency and blended methods of competency evaluation to determine the actual level of competency among nurses in integrating clients’ R/S into clinical practice. Finally, data collection was conducted one month after the intervention. Future longer follow-ups (3–6) are recommended to have more accurate results, determine the long-term impact of training, and assess the effect of educational courses on nurses’ competency in integrating clients’ R/S into clinical practice.

Given the strengths of the present study, our experiences in designing this online program can be beneficial for developing future educational programs aimed at fostering spiritual care competency and integrating clients’ R/S into clinical practice nurses. Moreover, Persian version of the RSIPAS, validated and translated in our study, can be applied in assessment and examination of other occupational groups such as social workers, educators’, clinical instructors’, and nursing students to assess their changes over time in Iranian context.

## Conclusions

Based on the findings, an online training program can empower nurses in integrating clients’ R/S into clinical practice. As a consequence, nurses are professionally and ethically responsible for providing spiritual care. Nurses’ higher competency levels toward integrating clients’ R/S into clinical practice and spiritual care can improve their healthcare interventions to provide holistic care for their clients. Our findings also highlighted the importance of applying initiative approaches to promote nurses’ competency in R/S issues. To this end, equipping nurses with spiritual care competencies and integration of clients’ R/S into clinical practice should start at the undergraduate level and continue with in-service education. In this regard, nursing professors are recommended to conduct systematic spiritual care training throughout the healthcare curriculum using new educational approaches, such as virtual (online and offline) and face-to-face teaching to overcome the practical gap in clients’ R/S integration. They also can benefit from our experiences in administration of the online training programs in nursing schools. Moreover, nurse mangers are suggested to plan in-service courses for training nurses in this regard. They should also provide opportunities to empower nurses for integrating clients’ R/S into clinical practice by motivating, creating organizational and managerial support programs, providing adequate manpower, reducing workload, creating space for interaction and problem-solving practices, and strengthening self-efficacy. Therefore, they can act more skillfully in holistic and comprehensive care programs to make more efficient decisions in clinical settings.

## Data Availability

The data are available upon request to the corresponding author after signing appropriate documents in line with ethical applications and decision of the Ethics Committee.

## Abbreviations

EBP: Evidence Based Practice
RSIPAS: Religious/Spiritually Integrated Practice Assessment Scale
R/S: religion/spirituality
